# Intensive care unit versus high-dependency care unit admission on mortality in patients with septic shock: a retrospective cohort study using Japanese claims data

**DOI:** 10.1186/s40560-022-00627-2

**Published:** 2022-07-22

**Authors:** Koji Endo, Kayoko Mizuno, Tomotsugu Seki, Woo Jin Joo, Chikashi Takeda, Masato Takeuchi, Koji Kawakami

**Affiliations:** 1 Department of Pharmacoepidemiology, Graduate School of Medicine and Public Health, Kyoto University, Yoshidakonoecho, Sakyo-ku, Kyoto, Japan; 2 Department of Cardiovascular Medicine, Graduate School of Medical Science, Kyoto Prefectural University of Medicine, 465 Kajii-cho, Kawaramachi-Hirokoji, Kamigyo-ku, Kyoto, Japan; 3 Department of Anesthesia, Kyoto University, Yoshidakonoecho, Sakyo-ku, Kyoto, Japan

**Keywords:** ICU, High-dependency care unit, Hospitalization, Mortality, Length of stay, Septic shock, Admission, Japan

## Abstract

**Background:**

Septic shock is a common and life-threatening condition that requires intensive care. Intensive care units (ICUs) in Japan are classified into ICUs and high-dependency care units (HDUs), depending on presence of full-time certified intensivists and the number of assigned nurses. Compared with other developed countries, there are fewer intensive care beds and certified intensivists in Japan; therefore, non-intensivists often treat patients with septic shock in HDUs. It is unknown where we should treat patients with septic shock because no studies have compared the clinical outcomes between ICU and HDU treatment. This study aimed to elucidate which units should admit patients with septic shock by comparing mortality data and resource use between ICU and HDU admissions.

**Methods:**

In this retrospective cohort study, we used a nationwide Japanese administrative database to identify adult patients with septic shock who were admitted to ICUs or HDUs between January 2010 and February 2021. The patients were divided into two groups, based on admittance to ICU or HDU on the day of hospitalization. The primary outcome was 30-day all-cause mortality adjusted for covariates using Cox regression analyses; the secondary outcomes were the length of ICU or HDU stay and length of hospital stay.

**Results:**

Of the 10,818 eligible hospitalizations for septic shock, 6584 were in the ICU group, and 4234 were in the HDU group. Cox regression analyses revealed that patients admitted to the ICUs had lower 30-day mortality (adjusted hazard ratio: 0.89; 95% confidence interval: 0.83–0.96; *P* = 0.005). Linear regression analyses showed no significant difference in hospital length of stay or ICU or HDU length of stay.

**Conclusions:**

An association was observed between ICU admission and lower 30-day mortality in patients with septic shock. These findings could provide essential insights for building a more appropriate treatment system.

## Background

Sepsis is a common and life-threatening disease with high mortality of 12.5–15%, so its disease burden is enormous [1, 2]. It is estimated that 47–50 million people worldwide, including children and those in developing countries, suffer from sepsis annually, and at least 11 million people die from sepsis [3]. It is also reported that more than 10,000 people die from sepsis every year in Japan [4].

Septic shock is defined as sepsis associated with circulatory and cellular metabolic abnormalities, and patients with septic shock have high hospital mortality [5]. Besides appropriate antimicrobial therapy and source control with drainage or surgery, intensive care with fluid resuscitation and vasopressors is the essential treatment strategy recommended by international guidelines for septic shock [6, 7]. In contrast, there is no clear recommendation or consensus on where to treat patients with septic shock. “Sepsis Treatment System” was first mentioned in the Japanese Clinical Practice Guidelines 2020 (J-SSCG 2020), which recommends that patients with sepsis who do not respond to initial fluid resuscitation be managed in units where intensive care can be provided [8]. However, no reports compare the clinical outcomes of patients admitted to intensive care units (ICUs) versus non-ICU settings; therefore, it is difficult to appropriately define the appropriate “units”.

Furthermore, although the overall number of beds per population is larger, there are several problems with intensive care in Japan, such as a smaller number of certified intensivists and intensive care beds than in other developed countries [9]. Therefore, even in the case of critical illnesses, such as septic shock, a certain number of patients are treated by non-intensivists in non-ICU settings in Japan. This study aimed to investigate the practice pattern for patients with septic shock in Japan and to elucidate which units should admit patients by comparing the mortality of ICU admission with high-dependency care unit (HDU) admission.

## Methods

### Overview of ICU and HDU system in Japan

There are three major categories of acute hospital beds in Japan depending on the patient-to-nurse ratio: ICUs, HDUs, and general wards (Table 1). Compared with HDUs, ICUs require more nurses and space, resulting in more expensive charges per admission. ICUs were further divided into two categories. ICUs for which “ICU management fee 1” can be charged are located in large, well-equipped hospitals such as university hospitals. They have higher standards for full-time staff and facilities: two or more certified intensivists, certified nurses, and certified clinical engineers. Conversely, the other category of ICUs requires a full-time physician, but not necessarily certified intensivists. HDUs and general wards do not require a full-time physician. In Japan, large hospitals often have both ICU and HDU, while middle-sized hospitals often have only ICU or HDU. Smaller community-based hospitals often do not have intensive care units. The Japanese government’s insurance policy limits ICU admission to critically ill patients, such as those with loss of consciousness, respiratory failure, and shock. However, the actual decision of admission to either ICUs or HDUs depends on the medical system of each region, availability of beds, and judgment of the attending physician. Therefore, critically ill patients in Japan are often admitted to HDUs in Japan.

**Table 1:** Categories of acute hospital beds in Japan

	Charges for admission	Patient–nurse ratio	Criteria
ICUs	ICU management fee 1	2:1	Full-time staff (two or more experienced certified intensivists, certified nurses, and clinical engineers)
	ICU management fee 2	2:1	Full-time staff (two or more experienced certified intensivists, certified nurses, and clinical engineers)
	Emergency and critical care unit management fee 2	2:1	Full-time physician (not necessary intensivists)
	Emergency and critical care unit management fee 4	2:1	Full-time physician (not necessary intensivists)
	ICU management fee 3	2:1	Full-time physician (not necessary intensivists)
	ICU management fee 4	2:1	Full-time physician (not necessary intensivists)
HDUs	Emergency and critical care unit management fee 1	4:1	Full-time physician (not necessary intensivists)
	Emergency and critical care unit management fee 3	4:1	Full-time physician (not necessary intensivists)
	High care unit management fee 1	4:1	No need for full-time physician
	High care unit management fee 2	5:1	No need for full-time physician
General wards		7:1	No need for full-time physician

### Study design and data source

We conducted a retrospective cohort study using the diagnostic procedure combination (DPC) database provided by Medical Data Vision Co., Ltd. (MDV; Tokyo, Japan) (MDV). This database has been used in previous epidemiological studies [10, 11]. DPC is a payment system for acute hospital inpatients, in which provider reimbursement is calculated based on a per-diem fee according to the diagnosis category [12].

The MDV database is fully anonymized and includes more than 35 million inpatient data points from 438 acute care hospitals, which account for approximately 25% of all hospitals that have opted for DPC (as of the end of April 2021). The database contains demographic data, medical and pharmacy claims data, clinical diagnoses, and medical procedures. The clinical diagnoses were recorded using the International Classification of Diseases, 10th revision (ICD-10) codes. Medical procedures were recorded using Japanese classification codes and medical billing codes. Unfortunately, this database does not include physiological data such as vital signs or information about hospitals in which each patient was hospitalized. The study protocol was approved by the Ethics Committee of Kyoto University Graduate School and Faculty of Medicine (R2653).

### Study participants

We identified patients with septic shock who were ≥ 18 years and admitted to ICUs or HDUs for intensive care on the day of hospitalization between January 2010 and February 2021. In this study, we defined patients who met the following criteria as having septic shock. First, patients with the diagnosis of both infection (ICD-10 codes A039, A021, A047, A207, A217, A227, A239, A241, A267, A280, A282, A327, A392, A393, A394, A400, A401, A402, A403, A408, A409, A410, A411, A412, A413, A414, A415, A418, A419, A427, B007, B377, J189, J440, N390) and organ dysfunction (ICD-10 codes J960, J969, J80, R092, R570, R571, R578, R579, I951, I959, N170, N171, N172, N178, N179, K720, K729, K763, F050, F059, G931, G934, G938, D695, D696, D65) were identified using the ICD-10 codes that matched the ICD-9 codes used in the previous validation study [13] (Table 2). These diagnoses were identified from the database as main diagnosis, admission diagnosis, diagnosis with the first or second highest medical costs, or comorbidity at admission. Second, patients in whom both intravenous antibiotics and noradrenaline were used on the day of hospitalization. If a patient was hospitalized more than once, we counted each hospitalization as a single hospitalization.

**Table 2:** ICD-10 codes used for inclusion criteria

Infection	
A039	Shigellosis, unspecified
A021	Salmonella sepsis
A047	Enterocolitis due to *Clostridium difficile*
A207	Septicemic plague
A217	Generalized tularemia
A227	Anthrax sepsis
A239	Brucellosis, unspecified
A241	Acute and fulminating melioidosis
A267	*Erysipelothrix* sepsis
A280	Pasteurellosis
A282	Extraintestinal yersiniosis
A327	Listerial sepsis
A392	Acute meningococcemia
A393	Chronic meningococcemia
A394	Meningococcemia, unspecified
A400	Sepsis due to *Streptococcus*, group A
A401	Sepsis due to *Streptococcus*, group B
A402	Sepsis due to *Streptococcus*, group D
A403	Sepsis due to *Streptococcus pneumoniae*
A408	Other streptococcal sepsis
A409	Streptococcal sepsis, unspecified
A410	Sepsis due to *Staphylococcus aureus*
A411	Other sepsis
A412	Sepsis due to unspecified *Staphylococcus*
A413	Sepsis due to *Haemophilus influenzae*
A414	Sepsis due to anaerobes
A415	Sepsis due to other Gram-negative organisms
A418	Other specified sepsis
A419	Sepsis, unspecified, includes: septicemia
A427	Actinomycotic sepsis
B007	Disseminated herpes viral disease, includes herpes viral sepsis
B377	Candidal sepsis
J189	Pneumonia, unspecified organism
J440	Chronic obstructive pulmonary disease with acute lower respiratory infection
N390	Urinary tract infection, site not specified

Organ dysfunction	
Respiratory	
J960	Acute respiratory failure
J969	Respiratory failure, unspecified
J80	Diseases of bronchus, not elsewhere classified
R092	Respiratory arrest
Cardiovascular	
R570	Cardiogenic shock
R571	Hypovolemic shock
R578	Other shock
R579	Shock, unspecified
I951	Orthostatic hypotension
I959	Hypotension, unspecified
Renal	
N170	Acute renal failure with tubular necrosis
N171	Acute renal failure with acute cortical necrosis
N172	Acute renal failure with medullary necrosis
N178	Other acute renal failure
N179	Acute renal failure, unspecified
Neurological	
K720	Acute and subacute hepatic failure
K729	Hepatic failure, unspecified
K763	Infarction of liver
F050	Delirium not superimposed on dementia, so described
F059	Delirium, unspecified
G931	Anoxic brain damage, not elsewhere classified
G934	Encephalopathy, unspecified
G938	Metabolic encephalopathy
Hematological	
D695	Secondary thrombocytopenia
D696	Thrombocytopenia, unspecified
D65	Disseminated intravascular coagulation (defibrination syndrome)

Patients who died within 24 h after hospitalization were excluded because they were probably so severely ill they would have died, regardless of the unit type. Patients with the following diseases and procedures at the time of hospitalization were also excluded because they may be incorrectly included by the criteria above: patients complicated with congestive heart failure (ICD-10 code I509), complicated with severe acute pancreatitis (ICD-10 code K859), and patients who underwent the following procedures: percutaneous coronary intervention, coronary artery bypass grafting, valve replacement, valvuloplasty, transcatheter aortic valve implantation, operation for aortic aneurysm or dissection, or endovascular aortic repair.

### Exposure and comparison

We defined patients admitted to the ICUs on the day of hospitalization as the exposure group and those admitted to HDUs on the day of hospitalization as the comparison group. We identified admission to the ICUs using Japanese claims codes (classification codes A3002, A3004, A3011, A3012, A3013, A3014) or the HDUs (classification codes A3001, A30011, A3003, A3004, A301-21, A301-22, A301-24). The claims codes present in both groups (classification code A3004) were further distinguished using the accompanying medical billing codes. We excluded the management fee for severe burns from A3003, A3004, A3012, and A3014 to exclude patients with severe burns. If patients with claims codes of both units were identified on the day of hospitalization, we considered them to belong to the first unit group where the initial location they were admitted prior to transfer.

### Outcomes

The primary outcome of this study was 30-day all-cause mortality. We identified patient deaths using the discharge outcomes recorded in the DPC database. The secondary outcomes were the length of ICU or HDU stay, length of hospital stay, discharge destination, and Barthel index at discharge. The Barthel index (BI) was calculated based on the activities of daily living (ADL) scores recorded in the DPC database. The cumulative BI score ranges from 0 to 100 points, with 0 indicating complete dependence in activities of daily living and 100 indicating complete independence.

### Covariates

The covariates for adjusting confounding factors were age, sex, Charlson comorbidity index [14], admission year, ambulance use, emergency charge, admission from the nursing home, and facility information, such as teaching hospital and number of hospital beds. The following procedures and treatments performed on the day of hospitalization were also identified from the database: emergency surgery or drainage procedures performed for infectious source control, mechanical ventilation, continuous renal replacement therapy, polymyxin B-immobilized fiber column direct hemoperfusion (PMX-DHP), venoatrial extracorporeal membrane oxygenation (VA-ECMO), use of two or more vasoactive agents (dopamine, noradrenaline, dobutamine, epinephrine, and vasopressin), blood transfusion (red blood cells, platelets, fresh frozen plasma), albumin preparations infusion, sedative drugs, narcotic drugs, recombinant thrombomodulin, antithrombin III preparations, low-dose glucocorticoids, and intravenous immunoglobulin. Each patient’s infection source was identified using ICD-10 codes recorded at admission, combined with emergency surgery or drainage procedures performed.

### Statistical analysis

Categorical and ordinal variables were summarized using numbers and percentages. If normally distributed continuous variables were summarized using mean and standard deviation, or median and interquartile range if not normally distributed.

We compared 30-day mortality between the ICU and HDU groups using the Kaplan–Meier method and log-rank test and estimated the hazard ratio using multivariable Cox proportional hazard models, adjusting for the covariates mentioned above. Patients who were transferred to other hospitals and discharged within 30 days of hospitalization were censored. The survival period was calculated from the date of hospitalization to the date of death from any cause within 30 days. Secondary outcomes were analyzed using a logistic regression model to evaluate the association between ICU admission and in-hospital mortality. A linear regression model was used to assess the length of ICU (or HDU) stay, length of hospital stay, and Barthel index on discharge. We adjusted all secondary outcomes for the same covariates as those in the survival analysis.

Subgroup analyses were performed for age, procedures performed on the day of hospitalization, and the source of infection. Sensitivity analyses were performed for limited populations as follows: (a) population which include the patients who met the exclusion criteria; (b) population with ICD-9 codes for infection and organ dysfunction from a previous study that did not match ICD-10 codes, supplemented with the corresponding ICD-10 codes (supplement ICD-9 codes: A41.50, A41.51, A41.52, A41.58 with ICD-10 code: A498, supplement ICD-9 code: R572 with ICD-10 code: A419); (c) admission to hospitals with both ICUs and HDUs; (d) 14-day all-cause mortality, and (e) in-hospital mortality. We conducted sensitivity analyses by changing the definition of exposure and comparison (f): (1) “ICU management fee 1” vs. “ICU management fee 3” and “Emergency and critical care unit management fee 2” to examine whether “ICU management fee 1” had better outcomes in ICUs; (2) “ICU management fee 1” vs. “Emergency and critical care unit management fee 1”, to compare outcomes for the most resource-rich ICUs and HDUs, respectively; (3) “ICU management fee 3” and “Emergency and critical care unit management fee 2” vs. “Emergency and critical care unit management fee 1”, to compare outcomes in more resource-poor ICUs to those in the most resource-rich HDUs. We also performed propensity score matching analyses using the nearest neighbor matching (g): (1) caliper width of 0.1 of the standard deviation; (2) caliper width of 0.2 of the standard deviation. A multivariable logistic regression model using all the covariates same as the primary analysis was employed to compute the propensity scores for patients admitted to the ICUs on the day of hospitalization. The statistical significance level was set at a two-tailed *p* < 0.05, and all statistical analyses were conducted using SAS ver. 9.4 (SAS Institute Inc., Cary, NC, USA).

## Results

**Fig. 1:**
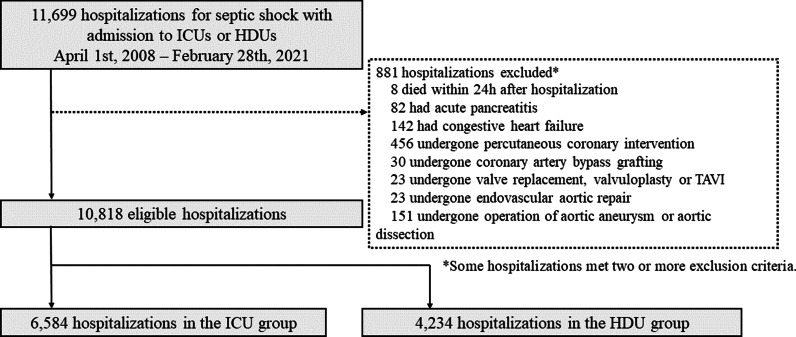
Study flow diagram

Overall, 11,699 hospitalizations of patients with septic shock and admissions to ICUs or HDUs were identified between April 2008 and February 2021 (Fig. 1). Of these, 881 hospitalizations met the exclusion criteria, and 10,818 eligible hospitalizations of 10,754 patients were included in the analysis. Of the included hospitalizations, 6584 (60.9%) were in the ICU group, and 4234 (39.1%) were in the HDU group. No patient was admitted to both ICU and HDU on the day of hospitalization. The baseline characteristics of the patients are presented in Table 3. Table 4 shows the treatments performed on the day of hospitalization. Although the baseline characteristics of both groups were similar, patients in the ICU group were more likely to receive intravenous drugs and interventions, such as mechanical ventilation, catheterization, or abdominal surgery.

**Table 3:** Baseline characteristics of patients

	Overall	ICU	HDU
	*n* = 10,818	*n* = 6584 (60.9%)	*n* = 4234 (39.1%)
Age (years), median (IQR)	76.0 (67.0–84.0)	75.0 (66.0–82.0)	78.0 (69.0–85.0)
Male sex, *n* (%)	6165 (57.0)	3813 (57.9)	2352 (55.6)
BMI^a^ (kg/m^2^), median (IQR)	21.3 (18.6–24.3)	21.5 (18.7–24.5)	21.1(18.3–24.0)
Charlson comorbidity index			
0, *n* (%)	8013 (74.1)	4857 (73.8)	3156 (74.5)
1, *n* (%)	1949 (18.0)	1181 (17.9)	768 (18.1)
≤ 2, *n* (%)	856 (7.9)	546 (8.3)	310 (7.3)
Source of infection			
Bacteremia/sepsis, *n* (%)	5228 (48.3)	3182 (48.3)	2046 (48.3)
Respiratory, *n* (%)	1635 (15.1)	971 (14.8)	664 (15.7)
Gastrointestinal, *n* (%)	1739 (16.1)	1293 (19.6)	446 (10.5)
Urinary tract, *n* (%)	1136 (10.5)	526 (8.0)	610 (14.4)
Hepatobiliary, *n* (%)	848 (7.8)	451 (6.9)	397 (9.4)
Skin/soft tissue, *n* (%)	174 (1.6)	123 (1.9)	51 (1.2)
Admission			
From home, *n* (%)	7265 (67.2)	4345 (66.0)	2920 (69.0)
From other hospital, *n* (%)	1793 (16.6)	1195 (18.2)	598 (14.1)
From nursing home, *n* (%)	852 (7.9)	374 (5.7)	478 (11.3)
Admission year			
2008–2012, *n* (%)	344 (3.2)	256 (3.9)	88 (2.1)
2013–2017, *n* (%)	4879 (45.1)	3117 (47.3)	1762 (41.6)
2018–2021, *n* (%)	5595 (51.7)	3211 (48.8)	2384 (56.3)
Ambulance use, *n* (%)	8870 (82.1)	5478 (83.4)	3392 (80.2)
Emergency charge, *n* (%)	5170 (47.8)	3145 (47.8)	2025 (47.8)
Hospital beds			
≤ 199, *n* (%)	112 (1.0)	58 (0.9)	54 (1.3)
200–499, *n* (%)	4796 (44.3)	2583 (39.2)	2213 (52.3)
≤ 500, *n* (%)	5910 (54.6)	3943 (59.9)	1967 (46.5)
Teaching hospital, *n* (%)	10,156 (93.9)	6233 (94.7)	3923 (92.7)

*IQR* interquartile range, *BMI* body mass index

^a^The number of patients missing BMI: ICU 649, HDU 543

**Table 4:** Treatment performed on the day of hospitalization

	Overall	ICU	HDU
	*n* = 10,818	*n* = 6584 (60.9%)	*n* = 4234 (39.1%)
Vasoactive agents			
Dopamine, *n* (%)	2168 (20.0)	1413 (21.5%)	755 (17.8)
Adrenaline, *n* (%)	982 (9.1)	720 (10.9%)	262 (6.2)
Dobutamine, *n* (%)	868 (8.0)	649 (9.9%)	219 (5.2)
Vasopressin, *n* (%)	1875 (17.3)	1479 (22.5%)	396 (9.4)
≥2 drugs, *n* (%)	4459 (41.2)	3146 (47.8%)	1313 (31.0)
≥3 drugs, *n* (%)	1193 (11.0)	924 (14.0%)	269 (6.4)
Transfusion			
Red blood cell, *n* (%)	1805 (16.7)	1404 (21.3)	401 (9.5)
Platelet, *n* (%)	667 (6.2)	521 (7.9)	146 (3.5)
Fresh frozen plasma, *n* (%)	1581 (14.6)	1300 (19.7)	281 (6.6)
Albumin, *n* (%)	3226 (29.8)	2403 (36.5)	823 (19.4)
Globulin, *n* (%)	1511 (14.0)	1105 (16.8)	406 (9.6)
Recombinant thrombomodulin, *n* (%)	2169 (20.1)	1281 (19.5)	888 (21.0)
Antithrombin III, *n* (%)	1108 (10.2)	827 (12.6)	281 (6.6)
Hydrocortisone, *n* (%)	2706 (25.0)	1922 (29.2)	784 (18.5)
Sedative drugs, *n* (%)	5796 (53.6)	4325 (65.7)	1471 (34.7)
Narcotic drugs, *n* (%)	4562 (42.2)	3481 (52.9)	1081 (25.5)
Prophylaxis of gastrointestinal ulcer, *n* (%)	6327 (58.5)	4470 (67.9)	1857 (43.9)
Prophylaxis of deep vein thrombosis, *n* (%)	3916 (36.2)	2762 (42.0)	1154 (27.3)
Rehabilitation, *n* (%)	516 (4.8)	469 (7.1)	47 (1.1)
Mechanical ventilation, *n* (%)	4049 (37.4)	2986 (45.4)	1063 (25.1)
CRRT, *n* (%)	1691 (15.6)	1351 (20.5)	340 (8.0)
PMX-DHP, *n* (%)	1025 (9.5)	746 (11.3)	279 (6.6)
VA-ECMO/IABP, *n* (%)	200 (1.9)	172 (2.6)	28 (0.7)
Central venous catheter, *n* (%)	7087 (65.5)	5028 (76.4)	2059 (48.6)
Arterial line, *n* (%)	6979 (64.5)	5159 (78.4)	1820 (43.0)
Urinary catheter, *n* (%)	8237 (76.1)	5109 (77.6)	3128 (73.9)
Nasogastric tube, *n* (%)	4449 (41.1)	3406 (51.7)	1043 (24.6)
Blood culture test, *n* (%)	9571 (88.5)	5799 (88.1)	3772 (89.1)
Antibiotics			
Penicillin, *n* (%)	3459 (32.0)	2087 (31.7)	1372 (32.4)
Cephalosporin, *n* (%)	2566 (23.7)	1554 (23.6)	1012 (23.9)
Carbapenem, *n* (%)	5896 (54.5)	3683 (55.9)	2213 (52.3)
Quinolone, *n* (%)	500 (4.6)	361 (5.5)	139 (3.3)
Anti-MRSA, *n* (%)	1484 (13.7)	1102 (16.7)	382 (9.0)
Aminoglycoside, *n* (%)	208 (1.9)	119 (1.8)	89 (2.1)
Metronidazole, *n* (%)	116 (1.1)	90 (1.4)	26 (0.6)
Anti-fungal, *n* (%)	180 (1.7)	137 (2.1)	43 (1.0)
Anti-viral, *n* (%)	248 (2.3)	176 (2.7)	72 (1.7)
Drainage			
Endoscopic, *n* (%)	357 (3.3)	178 (2.7)	179 (4.2)
Percutaneous, *n* (%)	214 (2.0)	127 (1.9)	87 (2.1)
Urinary, *n* (%)	754 (7.0)	388 (5.9)	366 (8.8)
Surgery			
Abdominal surgery, *n* (%)	1583 (14.6)	1207 (18.3)	376 (8.9)
Limb surgery, *n* (%)	92 (0.9)	67 (1.0)	25 (0.6)

*CRRT* continuous renal replacement therapy, *PMX-DHP* polymyxin B immobilized fiber column direct hemoperfusion, *VA-ECMO* venoatrial extracorporeal membrane oxygenation, *IABP* intra-aortic balloon pumping

**Fig. 2:**
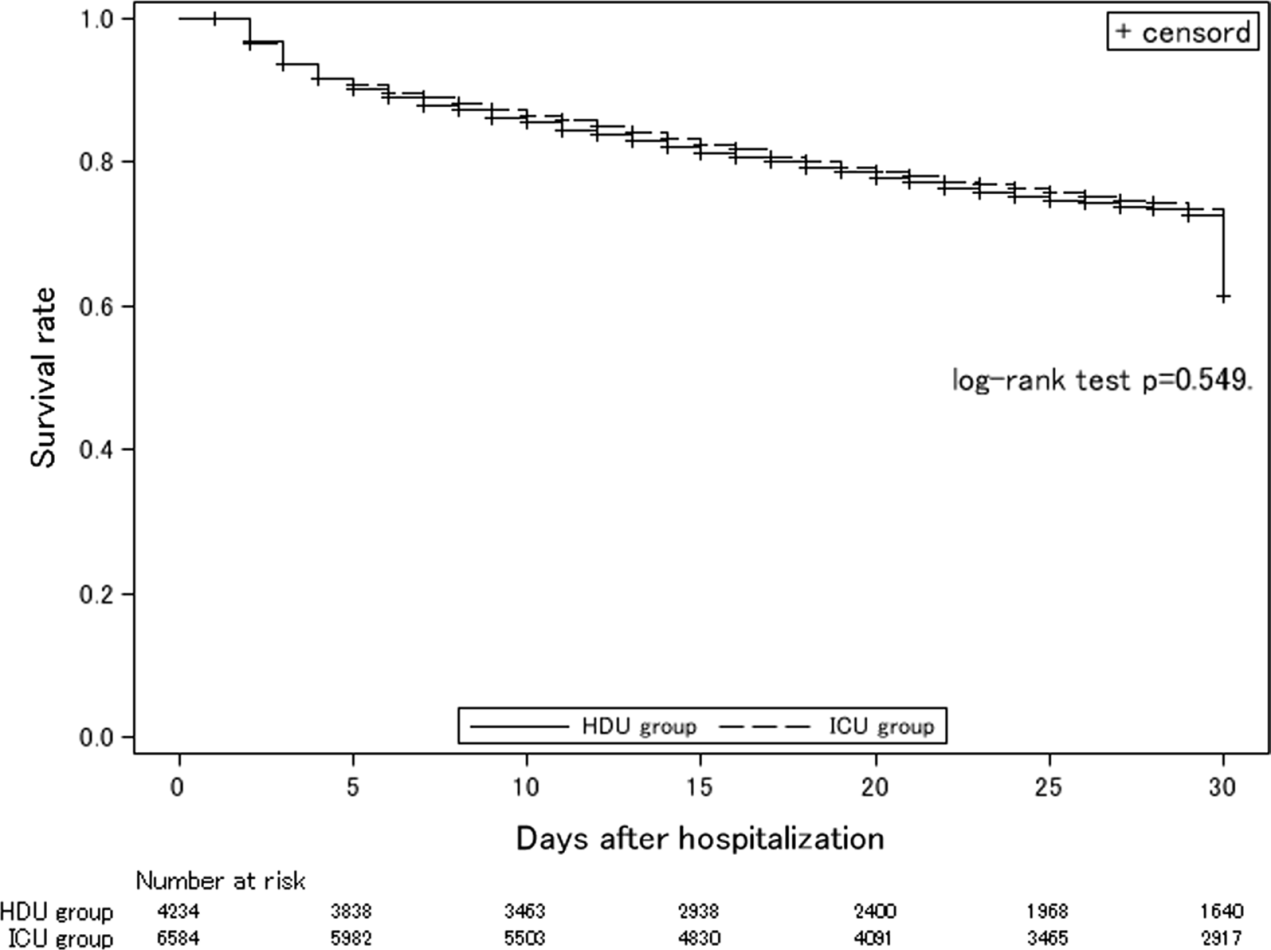
Survival analysis

Figure 2 shows the Kaplan–Meier survival plots for both groups. Cox regression analyses revealed that patients in the ICU group had lower 30-day mortality (adjusted hazard ratio: 0.89; 95% confidence interval 0.83–0.96; *p* = 0.005) (Table 5). The secondary outcomes are presented in Table 5. The incidence proportion of all-cause in-hospital death was approximately 30% in both groups. Logistic regression analysis showed that patients in the ICU group had lower in-hospital mortality than patients in the HDU group (adjusted odds ratio: 0.82; 95% confidence interval: 0.75–0.90; *p* < 0.001). Linear regression analyses showed no significant difference in hospital length of stay and ICU or HDU length of stay.

**Table 5:** Primary outcome and secondary outcomes

	Overall	ICU	HDU	Point estimates	95% CI	*P* value
Primary outcome						
30-day mortality	2602 (24.0)	1576 (23.9)	1026 (24.2)	0.89^a^	0.83–0.96	0.005
Secondary outcomes						
In-hospital death	3308 (30.6)	2041 (31.0)	1267 (29.9)	0.82^b^	0.75–0.90	< 0.001
Hospital length of stay, days	25.0 (13.0–46.0)	26.0 (14.0–48.0)	22.0 (12.0–43.0)	− 0.31^c^	− 1.92 to 1.28	0.69
ICU or HDU length of stay, days	6.0(3.0–13.0)	7.0(4.0–14.0)	5.0 (3.0–10.0)	0.11^c^	− 0.09 to 0.31	0.29
Discharge to home	3504 (32.4)	2036 (30.9)	1468 (34.7)	1.03^b^	0.94–1.14	0.42
Discharge to other hospitals	3501 (32.4)	2289 (34.8)	1212 (28.6)	1.20^b^	1.09–1.31	< 0.001
Discharge to nursing home	1310 (12.1)	812 (12.3)	498 (11.8)	1.12^b^	0.95–1.31	0.15
Barthel index on discharge^§^	50.0 (0.0–100.0)	50.0 (0.0–100.0)	45.0 (0.0–100.0)	2.32^c^	0.12–4.53	0.038

Data are presented as number of events (%) or mean (IQR)

*IQR* interquartile range, *CI* confidence interval, *CRRT* continuous renal replacement therapy, *PMX-DHP* polymyxin B immobilized fiber column direct hemoperfusion, *VA-ECMO* venoatrial extracorporeal membrane oxygenation, *IABP* intra-aortic balloon pumping

^§^The number of patients missing Barthel index: ICU 2499, HDU 1529

^a^Adjusted HR adjusted for age, sex, Charlson comorbidity index, admission year, ambulance use, teaching hospitals, emergency charge, hospital beds, patients from nursing home, source of infection, drainage, surgery, mechanical ventilation, CRRT, PMX-DHP, VA-ECMO, catecholamines, vasopressin, use of two or more catecholamines, transfusions (red blood cell, platelet, fresh frozen plasma), albumin, globulin, sedatives drugs, opioids drugs, recombinant thrombomodulin, antithrombin III, and hydrocortisone

^b^Adjusted odds ratio adjusted for the same covariates as *

^c^Regression coefficient adjusted for the same covariates as *

The results of subgroup and sensitivity analyses are shown in Table 6. Subgroup analyses showed that patients admitted to the ICU with VA-ECMO or with hepatobiliary diseases had significantly lower mortality. Sensitivity analyses performed in a population with different inclusion criteria also showed results consistent with those of the primary analysis. Fourteen-day and in-hospital all-cause mortality in Cox regression analyses were also lower in the ICU group. Cox regression analyses on propensity score-matched populations with different calipers also showed lower 30-day mortality in the ICU group.

**Table 6:** Subgroup analysis and sensitivity analysis

	30-day mortality			Adjusted HR	95% CI	*P* value
	Number of events/number of patients (%)^e^					
	Overall	ICU	HDU			
Subgroup analysis						
Age, years						0.71^§^
< 65	399/2149 (18.5)	282/1479 (19.0)	117/670 (17.4)	0.92^a^	0.74–1.13	0.440
65–74	607/2657 (22.8)	376/1679 (22.3)	231/978 (23.6)	0.83^a^	0.71–0.98	0.029
75–84	889/3679 (24.1)	540/2213 (24.4)	349/1466 (23.8)	0.93^a^	0.82–1.06	0.33
≥ 85	707/2333 (30.3)	378/1213 (31.1)	329/1120 (29.3)	0.92^a^	0.79–1.06	0.27
Procedures						
Mechanical ventilation	1266/4049 (31.2)	896/2986 (30.0)	370/1063 (34.8)	0.95^b^	0.85–1.07	0.44
CRRT	500/1691 (29.5)	403/1351 (29.8)	97/340 (28.5)	1.08^b^	0.89–1.33	0.44
PMX	257/1025 (25.0)	180/746 (24.1)	77/279 (27.6)	0.91^b^	0.7–1.19	0.52
VA-ECMO/IABP	67/200 (33.5)	51/172 (29.6)	16/28 (57.1)	0.35^b^	0.17–0.69	0.002
Source of infection						
Respiratory disease	442/1635 (27.0)	239/971 (24.6)	203/664 (30.5)	0.86^b^	0.71–1.03	0.1
Urinary tract disease	102/1136 (8.9)	50/526 (9.5)	52/610 (8.5)	1.06^b^	0.72–1.57	0.75
Gastrointestinal disease	379/1739 (21.7)	278/1293 (21.5)	101/446 (22.6)	1.10^b^	0.88–1.36	0.38
Hepatobiliary disease	128/848 (15.0)	61/451 (13.5)	67/397 (16.8)	0.68^b^	0.47–0.99	0.046
Skin/soft tissue	34/174 (19.5)	26/123 (21.1)	8/51 (15.6)	1.63^b^	0.72–3.71	0.23
Sensitivity analysis						
(a) population which include the patients who met the exclusion criteria	2859/11699 (24.4)	1759/7218 (24.3)	1100/4481 (24.5)	0.9^c^	0.84–0.97	0.008
(b) ICD-9 codes from the previous study supplemented with the corresponding ICD-10 codes	3204/13816 (23.1)	1965/8448 (23.2)	1239/5368 (23.0)	0.92^c^	0.86–0.98	0.02
(c) Hospital with ICUs and HDUs	1997/8311 (24.0)	1395/5798 (24.0)	602/2513 (23.9)	0.86^c^	0.78–0.95	0.002
(d) 14-day mortality	1800/10818 (16.6)	1068/6584 (16.2)	732/4234 (17.2)	0.88^c^	0.82–0.95	0.002
(e) In-hospital mortality	3308/10818 (30.5)	2041/6584 (31.0)	1267/4234 (29.9)	0.89^c^	0.83–0.96	0.005
(f) changing the definition of exposure and comparison^d^						
(1)	892/3539 (25.2)	167/671 (24.8)	725/2868 (25.2)	1.02^a^	0.86–1.21	0.78
(2)	589/2407 (24.4)	167/671 (24.8)	422/1736 (24.3)	0.86^a^	0.71–1.04	0.14
(3)	1147/4604 (24.9)	725/2868 (25.2)	422/1736 (24.3)	0.88^a^	0.77–1.00	0.052
(g) propensity score-matched population						
(1) caliper width of 0.1 of SD	1598/6788 (23.5)	746/3394 (21.9)	852/3394 (25.1)	0.89	0.82–0.97	0.013
(2) caliper width of 0.2 of SD	1765/7432 (23.7)	840/3716 (22.6)	925/3716 (24.8)	0.91	0.84–0.99	0.03

*CI* confidence interval, *SD* standard deviation, *CRRT* continuous renal replacement therapy, *PMX-DHP* polymyxin B immobilized fiber column direct hemoperfusion, *VA-ECMO* venoatrial extracorporeal membrane oxygenation; IABP, intra-aortic balloon pumping

^§^*P* for interaction

^a^Adjusted for sex, Charlson comorbidity index, admission year, ambulance use, teaching hospitals, emergency charge, hospital beds, patients from nursing home, source of infection, drainage, surgery, blood culture test, urinary chemistry test, mechanical ventilation, CRRT, PMX-DHP, VA-ECMO, catecholamines, vasopressin, use of two or more catecholamines, transfusions (red blood cell, platelet, fresh frozen plasma), albumin, globulin, sedatives drugs, opioids drugs, recombinant thrombomodulin, antithrombin III, and hydrocortisone

^b^Adjusted for age and the same covariates as *. Among these, procedure and source of infection that fell into each subgroup were excluded from the covariates

^c^Adjusted for age and the same covariates as *

^d^(1) “ICU management fee 1” vs. “ICU management fee 3” and “Emergency and critical care unit management fee 2”, (2) “ICU management fee 1” vs. “Emergency and critical care unit management fee 1”, (3) “ICU management fee 3” and “Emergency and critical care unit management fee 2” vs. “Emergency and critical care unit management fee 1”

^e^The “number of events” indicates deaths within 30 days of hospitalization, except for “14-day mortality”, which indicates deaths within 14 days

## Discussion

In this study, we investigated the association between ICU admission and 30-day mortality in patients with septic shock. In this large-scale cohort study in Japan, approximately 40% of the patients with septic shock were admitted to HDUs. Compared with HDU admission, ICU admission showed high-frequency administration of intravenous drugs, blood transfusions, and blood products. Moreover, the frequency of mechanical ventilation, renal replacement therapy, and device placement was also higher in the ICU group. Survival analysis showed that ICU admission had lower 30-day mortality than HDU admission. Subgroup analyses of patients who underwent VA-ECMO and those with hepatobiliary infections showed that ICU admission results in better outcomes than HDU admission. Sensitivity analyses also showed results consistent with the primary analysis.

These results provide valuable insights into the treatment locations of patients with septic shock. International guidelines for septic shock are based on studies that focus on ICU treatment, while treatment outside ICUs has not been well evaluated [6]. Therefore, there is only limited available evidence regarding where patients with septic shock should be treated. To the best of our knowledge, this is the first study to compare the outcomes of patients with septic shock in ICUs and HDUs.

Like our study, some observational studies using the Japanese DPC database have compared the clinical outcomes of ICUs and HDUs. Miki et al. reported that patients with acute myocardial infarction who were admitted to ICUs showed lower 30-day mortality than those admitted to non-ICUs [15]. Iwashita et al. also reported that the in-hospital mortality of patients who underwent mechanical ventilation in ICUs was lower than that of patients who underwent mechanical ventilation in HDUs [16]. In their study’s subgroup analysis of patients with sepsis, even though the ICU group had more patients requiring renal replacement therapy and device placement, suggesting that the severity of patients was higher in the ICU group, in-hospital mortality was also lower in the ICU group. In contrast, a recent propensity score-matched analysis by Ohbe et al. found that in-hospital mortality did not differ for patients with acute heart failure admitted to ICUs from that of patients admitted to high-dependency care units [17]. The reason for the better outcomes in the ICU group in the two previous studies and our study is unclear. However, it has been reported that patients treated in certified ICUs have better outcomes than those treated in non-certified ICUs and that high sepsis bundle adherence is associated with improved survival [18, 19]. Additionally, our sensitivity analysis by intensive care unit category showed that patients outcomes were better in the units with more nurses. Therefore, we can speculate that several factors influence these results: the number of intensivists and certified nurses, especially nursing staff, and the quality of care provided in the ICUs, including prevention of complications and high sepsis bundle compliance.

Based on the results of our study, it seems that patients with septic shock should be admitted to ICUs for intensive care. However, there are currently not enough intensivists and intensive care beds in Japan. Although the number of beds is rapidly increasing in response to the COVID-19 pandemic, the Japanese Society of Intensive Care Medicine reported that there were 7015 ICU beds and 13,003 HDU beds nationwide in 2020, with an overall intensive care bed count of approximately 15.9 beds per 100,000 population [20]. In contrast, the number of intensive care beds per 100,000 population in the United States and Germany was 34.7 (as of 2009) and 29.2 (as of 2010), respectively [21, 22]. Moreover, Japan has only 2115 certified intensivists (as of April 1, 2021) compared with approximately 12,000 certified intensivists in the United States. Further, intensivists in Japan tend to be unevenly distributed in some urban areas [20, 23]. Therefore, it is not feasible to treat all patients with sepsis in the ICUs.

To improve clinical outcomes in HDUs, we propose the following interventions for non-intensivists and non-certified nurses involved in intensive care: disseminate standardized treatments described in the guidelines, provide education using off-the-job training like Fundamental Critical Care Support (FCCS), and provide medical support systems such as tele-ICU. Subsequently, building a treatment system in each region should allow for better determination of which patients should be treated in the ICUs.

Our study had several limitations. First, some critical data were unavailable owing to the nature of the database designed for billing purposes. There is no information on the specialty of attending physicians, hospital volume, or commonly used severity scores based on physiological and laboratory parameters such as the Sequential Organ Failure Assessment (SOFA) score or Acute Physiology and Chronic Health Evaluation (APACHE) II score. These factors may be unmeasured confounders and influence the outcomes. In open ICUs, non-intensivists often provide treatment, while closed ICUs exist only in large hospitals, such as university hospitals. The database does not provide information on whether each patient was admitted to either open or closed ICUs. As mentioned above, some ICUs are staffed by certified intensivists; therefore, patients in closed ICUs are more likely to be treated by specialists. However, in other ICUs and HDUs, patients are more likely to be treated by nonspecialists. Furthermore, it has been reported that the hospital case sepsis volume influences the outcomes [24]. Since our database does not include information on where each patient was hospitalized, we do not have access to the annual number of cases. Compared to hospitals with only HDUs, case volume in hospitals with ICUs can be expected to be higher. In such cases, the effect of ICU admission on outcomes may be overestimated. Although we adjusted the severity with demographic data and the treatments performed at the time of hospitalization based on previous studies [17, 25], there may have been a difference in severity caused by these unmeasured confounders.

Second, misclassification of the included patients may have occurred due to uncertainty in the validity of the adopted ICD-10 codes as our eligibility criteria. These codes were partially modified versions of the ICD-9 codes used in a previous validation study, revealing that the sensitivity and specificity for severe sepsis (former criteria) in ICUs were 65% and 88%, respectively. Our study attempted to improve the validity by adding intravenous antibiotics and vasoactive agents to the inclusion criteria. Additionally, we performed several sensitivity analyses using different inclusion criteria and confirmed that the results were consistent. However, since there are no reports on diagnostic accuracy, the external validity of this study on the Japanese DPC database is unknown. The remaining uncertainty in the inclusion criteria is a major limitation of this study.

Third, there may be confounding by indication in selecting the treatment location. As previously described, large Japanese hospitals often have ICUs and HDUs. Whether patients are admitted to ICUs or HDUs is often determined by the admission rules of each hospital. For example, patients from the emergency room are admitted to the ICU, whereas patients after emergency surgery are admitted to the HDU. Although the database does not include information on hospitals where each patient was hospitalized, we obtained additional information from the MDV on whether the hospital had both ICU and HDU after 2018. This additional information is somewhat inaccurate because units may be converted during the observation period, resulting in the misclassification of ICUs and HDUs. However, a sensitivity analysis was performed on a limited population hospitalized in a hospital with both ICUs and HDUs, and the results were consistent with the primary analysis.

Fourth, there is a problem regarding non-informative censoring. In Japan, acute care hospitals are required to decrease hospital lengths of stay for bed control reasons, so patients whose conditions have stabilized are often transferred to skilled nursing and rehabilitation centers, particularly from inpatient units within large Japanese hospitals. Logistic regression analysis showed that patients in the ICU group were more frequently transferred to other hospitals than patients in the HDU group. This larger number of censored cases transferred to other hospitals in the ICU group may have missed deaths after transfer and resulted in an underestimation of mortality for the ICU group.

Finally, extrapolation of results should be performed with caution. The overall in-hospital mortality in both groups was approximately 30%, which is higher than the mortality reported in a previous study of patients with sepsis in Japan but consistent with that reported in a systematic review of patients with septic shock [26, 27]. We suspect this is because our inclusion criteria required the use of intravenous vasoactive agents to identify patients with shock and thus included patients with higher severity. Therefore, we suggest that the results of our study can only be extrapolated to critically ill patients with septic shock.

## Conclusions

In this retrospective cohort study, ICU admission for patients with septic shock was associated with lower mortality than HDU admission. Further investigations are required to develop an optimal sepsis treatment system.

## Data Availability

No data are available.

## Abbreviations

ICUs: Intensive care units
HDUs: High-dependency care units
DPC: Diagnostic procedure combination
BI: Barthel index
ADL: Activities of daily living
PMX-DHP: Polymyxin B-immobilized fiber column direct hemoperfusion
VA-ECMO: Venoatrial extracorporeal membrane oxygenation
FCCS: Fundamental critical care support
SOFA: Sequential Organ Failure Assessment
APACHE: Acute Physiology and Chronic Health Evaluation
