# Correction: Intensive care unit versus high-dependency care unit admission on mortality in patients with septic shock: a retrospective cohort study using Japanese claims data

**DOI:** 10.1186/s40560-022-00638-z

**Published:** 2022-10-12

**Authors:** Koji Endo, Kayoko Mizuno, Tomotsugu Seki, Woo Jin Joo, Chikashi Takeda, Masato Takeuchi, Koji Kawakami

**Affiliations:** 1 Department of Pharmacoepidemiology, Graduate School of Medicine and Public Health, Kyoto University, Yoshidakonoecho, Sakyo-ku, Kyoto, Japan; 2 Department of Cardiovascular Medicine, Graduate School of Medical Science, Kyoto Prefectural University of Medicine, 465 Kajii-cho, Kawaramachi-Hirokoji, Kamigyo-ku, Kyoto, Japan; 3 Department of Anesthesia, Kyoto University, Yoshidakonoecho, Sakyo-ku, Kyoto, Japan

## Correction: Journal of Intensive Care 10, 35 (2022) https://doi.org/10.1186/s40560-022-00627-2

Following publication of the original article [1], the authors reported three errors in Tables 5 and 6.

**Table 5:** Primary outcome and secondary outcomes

Primary outcome	Overall	ICU	HDU	Point estimates	95% CI	P value
30-day mortality	2602 (24.0)	1576 (23.9)	1026 (24.2)	0.89^a^	0.83–0.96	0.005
Secondary outcomes						
In-hospital death	3308 (30.6)	2041 (31.0)	1267 (29.9)	0.82^b^	0.75–0.90	< 0.001
Hospital length of stay, days	25.0 (13.0–46.0)	26.0 (14.0–48.0)	22.0 (12.0–43.0)	− 0.31^c^	− 1.92 to 1.28	0.69
ICU or HDU length of stay, days	6.0 (3.0–13.0)	7.0(4.0–14.0)	5.0 (3.0–10.0)	0.11^c^	− 0.09 to 0.31	0.29
Discharge to home	3504 (32.4)	2036 (30.9)	1468 (34.7)	1.03^b^	0.94–1.14	0.42
Discharge to other hospitals	3501 (32.4)	2289 (34.8)	1212 (28.6)	1.20^b^	1.09–1.31	< 0.001
Discharge to nursing home	1310 (12.1)	812 (12.3)	498 (11.8)	1.12^b^	0.95–1.31	0.15
Barthel index on discharge^§^	50.0 (0.0–100.0)	50.0 (0.0–100.0)	45.0 (0.0–100.0)	2.32^c^	0.12–4.53	0.038

Data are presented as number of events (%) or mean (IQR)

*IQR* interquartile range, *CI* confidence interval, *CRRT* continuous renal replacement therapy, *PMX-DHP* polymyxin B immobilized fiber column direct hemoperfusion, *VA-ECMO* venoatrial extracorporeal membrane oxygenation, *IABP* intra-aortic balloon pumping

^§^The number of patients missing Barthel index: ICU 2499, HDU 1529

^a^Adjusted HR adjusted for age, sex, Charlson comorbidity index, admission year, ambulance use, teaching hospitals, emergency charge, hospital beds, patients from nursing home, source of infection, drainage, surgery, mechanical ventilation, CRRT, PMX-DHP, VA-ECMO, use of two or more catecholamines, transfusions (red blood cell, platelet, fresh frozen plasma), albumin, globulin, sedatives drugs, opioids drugs, recombinant thrombomodulin, antithrombin III, and hydrocortisone

^b^Adjusted odds ratio adjusted for the same covariates as ^a^

^c^Regression coefficient adjusted for the same covariates as ^a^

**Table 6:** Subgroup analysis and sensitivity analysis

	30-day mortality					
	Number of events/number of patients (%)^e^					
	Overall	ICU	HDU	Adjusted HR	95% CI	P value
Subgroup analysis						
Age, years						0.71^§^
< 65	399/2149 (18.5)	282/1479 (19.0)	117/670 (17.4)	0.92^a^	0.74–1.13	0.440
65–74	607/2657 (22.8)	376/1679 (22.3)	231/978 (23.6)	0.83^a^	0.71–0.98	0.029
75–84	889/3679 (24.1)	540/2213 (24.4)	349/1466 (23.8)	0.93^a^	0.82–1.06	0.33
≥ 85	707/2333 (30.3)	378/1213 (31.1)	329/1120 (29.3)	0.92^a^	0.79–1.06	0.27
Procedures						
Mechanical ventilation	1266/4049 (31.2)	896/2986 (30.0)	370/1063 (34.8)	0.95^b^	0.85–1.07	0.44
CRRT	500/1691 (29.5)	403/1351 (29.8)	97/340 (28.5)	1.08^b^	0.89–1.33	0.44
PMX	257/1025 (25.0)	180/746 (24.1)	77/279 (27.6)	0.91^b^	0.7–1.19	0.52
VA-ECMO/IABP	67/200 (33.5)	51/172 (29.6)	16/28 (57.1)	0.35^b^	0.17–0.69	0.002
Source of infection						
Respiratory disease	442/1635 (27.0)	239/971 (24.6)	203/664 (30.5)	0.86^b^	0.71–1.03	0.1
Urinary tract disease	102/1136 (8.9)	50/526 (9.5)	52/610 (8.5)	1.06^b^	0.72–1.57	0.75
Gastrointestinal disease	379/1739 (21.7)	278/1293 (21.5)	101/446 (22.6)	1.10^b^	0.88–1.36	0.38
Hepatobiliary disease	128/848 (15.0)	61/451 (13.5)	67/397 (16.8)	0.68^b^	0.47–0.99	0.046
Skin/soft tissue	34/174 (19.5)	26/123 (21.1)	8/51 (15.6)	1.63^b^	0.72–3.71	0.23
Sensitivity analysis						
(a) population which include the patients who met the exclusion criteria	2859/11699 (24.4)	1759/7218 (24.3)	1100/4481 (24.5)	0.9^c^	0.84–0.97	0.008
(b) ICD-9 codes from the previous study supplemented with the corresponding ICD-10 codes	3204/13816 (23.1)	1965/8448 (23.2)	1239/5368 (23.0)	0.92^c^	0.86–0.98	0.02
(c) Hospital with ICUs and HDUs	1997/8311 (24.0)	1395/5798 (24.0)	602/2513 (23.9)	0.86^c^	0.78–0.95	0.002
(d) 14-day mortality	1800/10818 (16.6)	1068/6584 (16.2)	732/4234 (17.2)	0.88^c^	0.82–0.95	0.002
(e) In-hospital mortality	3308/10818 (30.5)	2041/6584 (31.0)	1267/4234 (29.9)	0.89^c^	0.83–0.96	0.005
(f) changing the definition of exposure and comparison^d^						
(1)	892/3539 (25.2)	167/671 (24.8)	725/2868 (25.2)	1.02^a^	0.86–1.21	0.78
(2)	589/2407 (24.4)	167/671 (24.8)	422/1736 (24.3)	0.86^a^	0.71–1.04	0.14
(3)	1147/4604 (24.9)	725/2868 (25.2)	422/1736 (24.3)	0.88^a^	0.77–1.00	0.052
(g) propensity score-matched population						
(1) caliper width of 0.1 of SD	1598/6788 (23.5)	746/3394 (21.9)	852/3394 (25.1)	0.89	0.82–0.97	0.013
(2) caliper width of 0.2 of SD	1765/7432 (23.7)	840/3716 (22.6)	925/3716 (24.8)	0.91	0.84–0.99	0.03

*CI* confidence interval, *SD* standard deviation, *CRRT* continuous renal replacement therapy, *PMX-DHP* polymyxin B immobilized fiber column direct hemoperfusion, *VA-ECMO* venoatrial extracorporeal membrane oxygenation, *IABP* intra-aortic balloon pumping

^§^*P* for interaction

^a^Adjusted for sex, Charlson comorbidity index, admission year, ambulance use, teaching hospitals, emergency charge, hospital beds, patients from nursing home, source of infection, drainage, surgery, mechanical ventilation, CRRT, PMX-DHP, VA-ECMO, use of two or more catecholamines, transfusions (red blood cell, platelet, fresh frozen plasma), albumin, globulin, sedatives drugs, opioids drugs, recombinant thrombomodulin, antithrombin III, and hydrocortisone

^b^Adjusted for age and the same covariates as ^a^. Among these, procedure and source of infection that fell into each subgroup were excluded from the covariates

^c^Adjusted for age and the same covariates as ^a^

^d^(1) “ICU management fee 1” vs. “ICU management fee 3” and “Emergency and critical care unit management fee 2”, (2) “ICU management fee 1” vs. “Emergency and critical care unit management fee 1”, (3) “ICU management fee 3” and “Emergency and critical care unit management fee 2” vs. “Emergency and critical care unit management fee 1”

^e^The "number of events" indicates deaths within 30 days of hospitalization, except for "14-days mortality," which indicates deaths within 14 days

In the annotation “a” of Table 5, “catecholamines” and “vasopressin” have been mistakenly listed as covariates and should be removed.

The correct annotation “a” of Table 5 should read: “Adjusted HR adjusted for age, sex, Charlson comorbidity index, admission year, ambulance use, teaching hospitals, emergency charge, hospital beds, patients from nursing home, source of infection, drainage, surgery, mechanical ventilation, CRRT, PMX-DHP, VA-ECMO, use of two or more catecholamines, transfusions (red blood cell, platelet, fresh frozen plasma), albumin, globulin, sedatives drugs, opioids drugs, recombinant thrombomodulin, antithrombin III, and hydrocortisone”.

In the annotation “a” of Table 6, “blood culture test”, “urinary chemistry test”, “catecholamines” and “vasopressin” have been mistakenly listed as covariates and should be removed.

The correct annotation “a” of Table 6 should read: “Adjusted for sex, Charlson comorbidity index, admission year, ambulance use, teaching hospitals, emergency charge, hospital beds, patients from nursing home, source of infection, drainage, surgery, mechanical ventilation, CRRT, PMX-DHP, VA-ECMO, use of two or more catecholamines, transfusions (red blood cell, platelet, fresh frozen plasma), albumin, globulin, sedatives drugs, opioids drugs, recombinant thrombomodulin, antithrombin III, and hydrocortisone”.

In addition, the “*” in annotation b and annotation c of both Tables 5 and 6 should be replaced by “a”.

The correct annotation b of Table 5 should read: “Adjusted odds ratio adjusted for the same covariates as a”.

The correct annotation c of Table 5 should read: “Regression coefficient adjusted for the same covariates as a”.

The correct annotation b of Table 6 should read: “Adjusted for age and the same covariates as a. Among these, procedure and source of infection that fell into each subgroup were excluded from the covariates”.

The correct annotation c of Table 6 should read: “Adjusted for age and the same covariates as a”.

The original article [1] has been updated.
